# The rs1800470 Polymorphism of the TGFB1 Gene Is Associated with Myocardial Fibrosis in Heart Transplant Recipients

**DOI:** 10.32607/actanaturae.11469

**Published:** 2021

**Authors:** O. E. Gichkun, O. P. Shevchenko, R. M. Kurabekova, N. P. Mozheiko, A. O. Shevchenko

**Affiliations:** Shumakov National Medical Research Center of Transplantology and Artificial Organs, Moscow, 123182 Russia; I.M. Sechenov First Moscow State Medical University (Sechenov University), Moscow, 119435 Russia

**Keywords:** transforming growth factor β1 gene, single nucleotide polymorphisms, myocardial fibrosis, cardiac, allograft

## Abstract

The transforming growth factor β1 (TGFβ1), whose level may depend on the polymorphism of the TGFB1 gene, is involved in the formation of myocardial fibrosis. Myocardial fibrosis in a cardiac allograft may lead to a heart’s structural and functional remodeling and subsequent dysfunction. The frequency of occurrence of alleles and genotypes of the TGFB1 gene polymorphic regions rs1800469, rs1800470, and rs1800471 in heart transplant recipients and their association with graft myocardial fibrosis were analyzed. Carriers of the CC genotype (p = 0.023, OR = 0.12, 95% CI: 0.017–1.0), and more often the G allele of rs1800471 (p = 0.023, OR = 7.76, 95% CI: 1.0–60.20), were found among heart transplant recipients less frequently than among healthy individuals. In patients with ischemic heart disease (IHD), the GG genotype was less common (p = 0.035, OR = 2.68, 95% CI: 1.061–6.793), while the A allele of rs1800469 was found more frequently (p = 0.035, OR = 0.37 95% CI: 0.148–0.942) than in patients with dilated cardiomyopathy (DCM). In heart transplant recipients with the AA genotype of rs1800470, myocardial fibrosis, verified by endomyocardial biopsy, was detected more often than in carriers of the G allele (OR = 10.4, 95% CI: 1.152–94.538, p = 0.013). The revealed differences suggest a relationship between TGFB1 gene polymorphism and graft myocardial fibrosis. Studies on a larger group of patients would make it possible to characterize the influence of genetic factors on the formation of myocardial fibrosis in heart transplant recipients.

## INTRODUCTION

The number of patients with heart failure is constantly on the rise as the mean life expectancy increases, while mortality due to acute medical conditions in young and middle-aged individuals declines. Heart transplantation is an efficient method for treating end-stage heart failure that improves the prognosis and quality of life of patients. The recently observed increase in the longevity of heart transplant recipients has been achieved mainly thanks to a reduction in the mortality rate during the early posttransplant period. Myocardial fibrosis, whose development is accompanied by structural and functional remodeling of the cardiac graft, is among the factors with an unfavorable impact on the long-term outcome of heart transplantation [1].

Graft myocardial fibrosis is a multifactorial process, with a number of cellular and molecular factors predisposing one to it [2]. Recent studies have shown that transforming growth factor β1 (TGFβ1), a profibrotic mediator involved in the production of the extracellular matrix, is among the pathogenetic factors of fibrosis [3].

The finding that the TGFβ1 level in the peripheral blood is genetically determined is now a fact. Some genetic polymorphisms of the TGFB1 gene were shown to be associated with the severity of coronary artery atherosclerosis and genetic predisposition to myocardial infarction; this association varies for different ethnic groups [4, 5].

The TGFβ1 protein is encoded by the TGFB1 gene residing on chromosome 19. Eight single nucleotide polymorphisms and a deletion/insertion polymorphism affecting the expression and activity of TGFβ1 have been identified thus far [6]. Researchers put particular focus on three TGFB1 gene polymorphisms associated with cardiovascular diseases: rs1800469 is localized in the promoter region, and rs1800470 (leucine-to-proline substitution in codon 10) and rs1800471 (arginine-to-proline substitution in codon 25) are localized in the coding region. Data on the effect of TGFB1 gene polymorphisms on the long-term outcomes of heart transplantation and genetic predisposition to developing post-transplant complications – acute and chronic (cardiac allograft vasculopathy) transplant rejection – are scarce and far from definitive [7, 8, 9].

This study aims at uncovering any association between the rs1800469, rs1800470, and rs1800471 polymorphisms of the TGFB1 gene and myocardial fibrosis in cardiac allograft in heart transplant recipients.

## EXPERIMENTAL

A total of 110 randomly selected heart transplant recipients who had undergone cardiac allograft transplantation at the Shumakov National Medical Research Center of Transplantology and Artificial Organs in 2017–2019 were enrolled in the study. All study participants were ethnic Russians; of those, 99 (84%) patients were males: the recipients’ mean age was 44 ± 14 (range: 16–70) years. The reason for the development of the end-stage heart failure responsible for the indications to transplantation was dilated cardiomyopathy (DCM) in 57 patients and ischemic heart disease (IHD) in 53 patients. The duration of the follow-up period after heart transplantation extended up to 4 (2.3 ± 1.3) years.

The patients were examined and treated in compliance with the clinical guidelines of the Russian Transplant Society. Endomyocardial biopsy for heart transplant recipients was performed according to the protocol during scheduled clinical laboratory examination or if there were respective indications. Endomyocardial biopsy specimens were assessed based on the histological and immunohistochemical data. Thin sections of endomyocardial tissue were subjected to Masson’s trichrome staining to confirm fibrosis in a cardiac allograft [10].

Genomic DNA was isolated from peripheral blood in accordance with the protocol, using a commercial QIAamp DNA Blood Mini Kit on a QIAcube™ automated analyzer (Qiagen, Germany). The rs1800469, rs1800470, and rs1800471 polymorphisms of the TGFB1 gene were analyzed by real-time polymerase chain reaction using TaqMan probes (Applied Biosystems, USA) on a CFX96^™^ amplification system (Bio-Rad, USA). The probes fluorescently labeled using VIC (allele 1)/FAM (allele 2) channels were detected at each amplification cycle. The resulting data were analyzed using the BioRad CFX manager 3.0 software.

The statistical analysis was carried out using the suite of applied software for research and engineering computations IBM SPSS STATISTICS 20 (IBM SPSS Inc., USA). In order to prove an independent distribution of alleles in the analyzed polymorphisms, their compliance to the Hardy–Weinberg Equilibrium was tested [11]. The frequencies of the genotypes or individual alleles in different groups were compared using the Pearson’s χ2 test. The potential effect of the genotype on a trait was assessed by determining the odds ratio and 95% confidence intervals. The critical value for the significance level was assumed to be 0.05.

## RESULTS

Genomic typing of the rs1800469, rs1800470, and rs1800471 polymorphisms of the TGFB1 gene in heart transplant recipients was conducted. No deviations in the distribution of the alleles and genotypes from the Hardy–Weinberg Equilibrium were revealed in the sex- and age-matched control group, consisting of healthy individuals (43). Compliance with the Hardy–Weinberg Equilibrium was detected in none of the heart transplant recipients for all three polymorphisms (Table 1).

**Table 1:** Data analysis for compliance with the Hardy– Weinberg equilibrium

Groups \ SNP	rs1800469	rs1800470	rs1800471
Healthy individuals	χ^2^ = 1.0 p = 0.31	χ^2^ = 0.02 p = 0.81	χ^2^ = 0.006 p = 0.93
Heart transplant recipients	χ^2^ = 4.32 p = 0.03*	χ^2^ = 9.3 p = 0.002*	χ^2^ = 5.73 p = 0.01*

^*^p < 0.05 – does not comply with the Hardy–Weinberg.

SNP – single nucleotide polymorphism.

The deviation from the Hardy–Weinberg Equilibrium in the heart transplant recipient group can be associated with a cardiovascular pathology, although the effect of a small sample size should not be ruled out; so, a larger sample is needed for testing.

**Table 2:** Distribution of genotypes and alleles of the polymorphic regions of the TGFB1 gene in heart transplant recipients and healthy individuals

Genotype/allele	Heart transplant recipients, n (%)	Healthy individuals, n (%)	p
rs1800469			
AA AG GG A G	22(20) 42 (38) 46 (42) 64 (58) 88 (80)	6(14) 16 (37) 21 (49) 22 (51) 37 (86)	0.38 0.91 0.43 0.43 0.38
rs1800470			
AA AG GG A G	91 (83) 14 (13) 4 (4) 105 (96) 18 (17)	40 (93) 3 (7) - 43 (100) 3 (7)	0.12 0.30 0.20 0.20 0.12
rs1800471			
GG GC CC G C	3 (3) 14 (13) 92 (84) 17 (16) 106 (97)	- 1 (2) 42 (98) 1 (2) 43 (100)	0.27 0.051 0.023* 0.023* 0.27

^*^*p < 0.05.

Table 2 shows the distribution of alleles and genotypes of the TGFB1 gene in heart transplant recipients and in the control group.

**Fig. 1:**
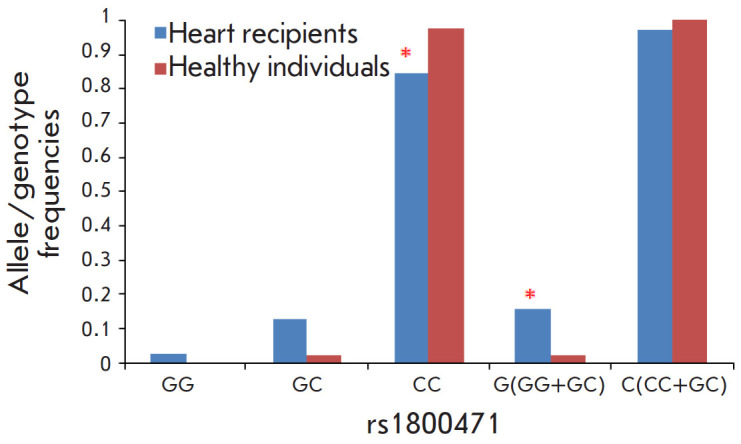
Frequency distribution of the alleles and genotypes of the rs1800471 polymorphism of the *TGFB1* gene in heart transplant recipients and healthy individuals. *p < 0.05 compared to healthy individuals

A comparative analysis of the distribution of the genotypes and alleles of the rs1800469 and rs1800470 polymorphisms of the TGFB1 gene detected no differences between healthy individuals and heart transplant recipients. However, some differences in the distribution of the alleles and genotypes of the rs1800471 polymorphism of the TGFB1 gene were found (Fig. 1).

Carriers of the CC genotype of the rs1800471 polymorphism of the TGFB1 gene were found among heart transplant recipients less frequently than among healthy individuals (p = 0.023; OR = 0.12; 95% CI, 0.017–1.0), while carriers of the G allele were found more often (within the GG and GC genotypes) (p = 0.023; OR = 7.76; 95% CI, 1.0–60.2).

An analysis of the association between the frequencies of distribution of the studied polymorphisms and the demographic and clinical characteristics of the heart transplant recipients revealed statistically significant associations with sex, diagnosis, and myocardial fibrosis in a cardiac allograft.

**Fig. 2:**
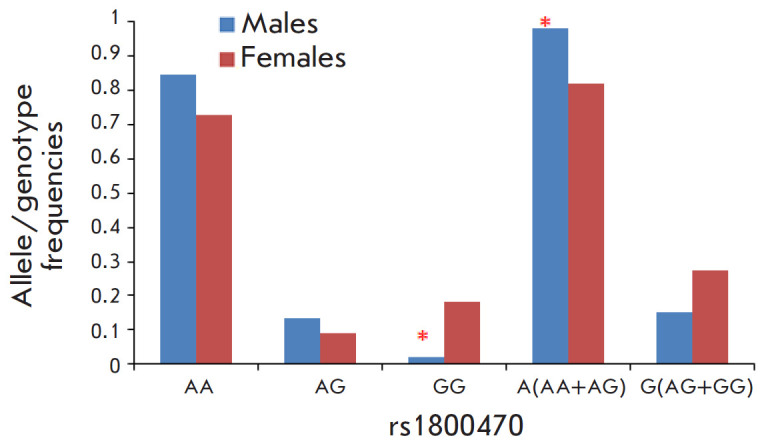
Frequency distribution of the alleles and genotypes of the rs1800470 polymorphism of the *TGFB1 *gene in males and females, *p < 0.05 compared to females

The distribution of the alleles and genotypes of the rs1800470 polymorphism of the TGFB1 gene differed significantly among male and female heart transplant recipients (Fig. 2).

Male heart transplant recipients were less likely to carry the GG genotype than females (p ≤ 0.05). A comparison of the frequencies of individual alleles showed that the frequency of the A rs1800470 allele of the TGFB1 gene is higher in males (p = 0.007; OR = 10.6; 95% CI, 1.34–85.01). No differences in the distribution of the genotypes and alleles of the rs1800469 and rs1800471 polymorphisms of the TGFB1 gene among males and females were revealed.

**Fig. 3:**
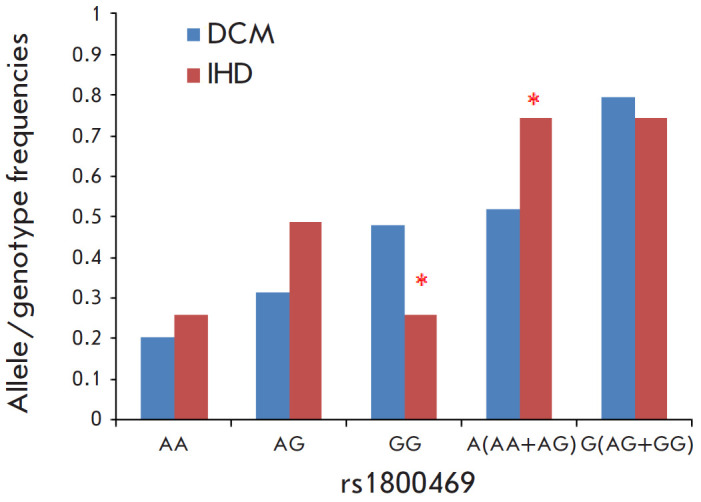
Frequency distribution of the alleles and genotypes of the rs1800469 polymorphism of the *TGFB1 *gene in patients with DCM and IHD, *p < 0.05 compared to DCM

A comparison analysis of the frequencies of the alleles and genotypes of the polymorphisms in the TGFB1 gene depending on the disease responsible for the heart failure and subsequent cardiac allograft transplantation revealed that patients with DCM carried the GG genotype of rs1800469 more frequently than patients with IHD did (p = 0.03; OR = 2.68; 95% CI, 1.061–6.793) (Fig. 3).

The frequency of the A allele among patients with IHD was higher than that among patients with DCM (p = 0.01, OR = 0.37; 95% CI, 0.148–0.942).

Examination of the endomyocardial biopsy specimens showed that myocardial interstitial fibrosis in cardiac allografts was verified in 49 out of 110 transplant recipients. Staining made it possible to clearly discern the connective tissue, which was colored in various shades of blue (depending on its maturation state) and differed from other cardiac muscle tissues. All fibrosis types (diffuse, focal, and diffuse/focal) were taken into account during the analysis.

**Fig. 4:**
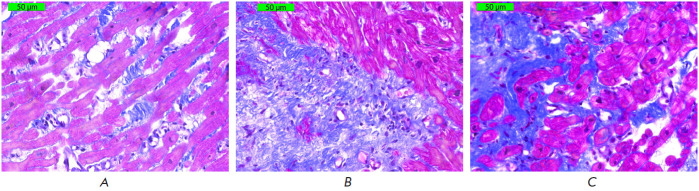
Histological preparations of endomyocardial biopsy specimens. Masson’s trichrome staining 400× (connective tissue is stained blue; cardiomyocytes are stained pink). (*A*) – diffuse overgrowth of loose fibrous connective tissue with single fibroplastic cells, focal granular proteinaceous degeneration of cardiomyocytes. (*B*) – focal overgrowth of non-mature connective tissue with single connective tissue cells, moderate proteinaceous degeneration of cardiomyocytes. (*C*) – diffuse/focal overgrowth of loose fibrous connective tissue, where proliferation of connective tissue cells is detected. Focal proteinaceous degeneration of cardiomyocytes

Figure 4 shows the histological preparations of cardiac allograft biopsy specimens with fibrotic changes.

**Fig. 5:**
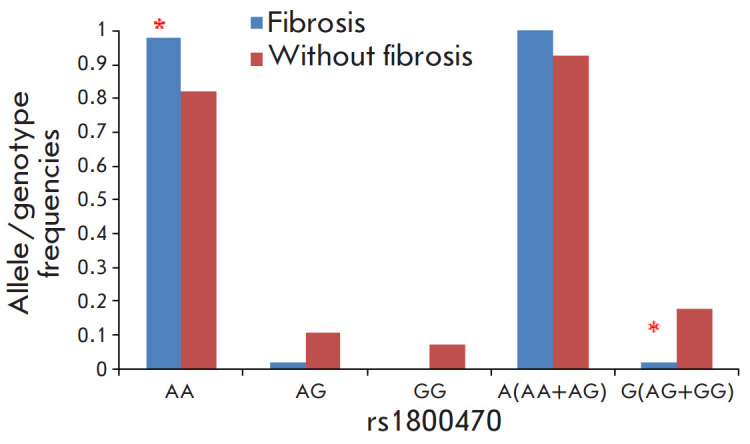
Frequency distribution of the alleles and genotypes of the rs1800470 polymorphism of the *TGFB1 *gene in heart transplant recipients with and without graft myocardial fibrosis, *p < 0.05 compared to heart transplant recipients without fibrosis

A comparative analysis of the frequency and genotype distribution revealed differences in the frequency of the AA genotype of the rs1800470 polymorphism of the TGFB1 gene in heart transplant recipients with and without myocardial fibrosis (Fig. 5).

Heart transplant recipients carrying the AA genotype of the rs1800470 polymorphism of the TGFB1 gene were more likely to have fibrosis than those carrying the G allele (OR = 10.4; 95% CI, 1.152–94.538, p = 0.013).

## DISCUSSION

This study has analyzed the distribution of the alleles and genotypes of three functionally significant polymorphisms of the TGFB1 gene in heart transplant recipients. In heart transplant recipients, differences in the frequency of occurrence of genotypes and alleles of the rs1800471 polymorphism of the TGFB1 gene were found in comparison with healthy individuals. A number of studies have shown that the G allele of the rs1800471 polymorphism is associated with a higher level of gene expression and elevated blood level of TGFβ1. Dysregulation of the TGFβ1 signaling pathway caused by a mutation can be associated with an increased risk of cardiovascular diseases [12, 13].

When analyzing the genetic predisposition to the primary disease that had been responsible for the end-stage heart failure and had made it necessary to perform heart transplantation, we observed a significant association between ischemic heart disease and the rs1800469 single nucleotide polymorphism. Similar data were obtained by Barsova et al. [4], who found a positive association between the TGFB1*-509T (rs1800469) allele and genetic predisposition to early myocardial infarction (in patients younger than 50 years). It still remains unclear what are the mechanisms underlying the association between the polymorphism and the development of end-stage heart failure. It is possible that the rs1800469 polymorphism alters promoter affinity for the transcription factors and inhibits TGFβ expression, thus activating proinflammatory cytokines (tumor necrosis factor α and interleukin-1), which may contribute to the progression of IHD [4]. Inconclusive data have been obtained in a number of studies: thus, no association between the 509C/T polymorphism and IHD was detected in a group of German patients [14]. Liu et al. [15] performed a meta-analysis of eight studies and showed a statistically significant association between the rs1800469 (TT) polymorphism and an increased risk of IHD.

Having analyzed the genetic predisposition to myocardial fibrosis in heart transplant recipients, we found significant associations between this trait and the rs1800470 polymorphism. The A allele of this polymorphism is known to be associated with a high level of TGFβ1 in peripheral blood. TGFβ1 is a potent stimulator of extracellular matrix production; its hyperproduction is associated with fibrotic disorders and the development of myocardial fibrosis. Leask [16] showed that TGFβ added to a fibroblast culture in vitro induces the expression of the genes related to extracellular matrix production and thus increases matrix accumulation and contributes to a concomitant suppression of matrix metalloproteinase production by raising the level of inhibitors of the gene encoding its expression.

## CONCLUSIONS

This study has revealed the differences in occurrence of the alleles and genotypes of the TGFB1 gene: the rs1800471 polymorphism in heart transplant recipients and healthy individuals, the rs1800469 polymorphism in patients with DCM and IHD, and the rs1800470 polymorphism in patients with and without myocardial fibrosis in a cardiac allograft. The findings give grounds for assuming that the TGFB1 gene and its polymorphic variants are involved in the formation of genetic predisposition to myocardial fibrosis in heart transplant recipients. Further studies using a larger cohort of patients would provide more specific characteristics of the impact of single nucleotide polymorphisms on the development of fibrosis in a cardiac allograft.

## Abbreviations

TGFβ1: transforming growth factor β1
TGFB1: the gene encoding TGF β1
DCM: dilated cardiomyopathy
IHD: ischemic heart disease
SNP: single nucleotide polymorphism
OR: odds ratio
PCR: polymerase chain reaction
